# LEVERAGING VIDEO TECHNOLOGY TO DELIVER SOCIAL ENGAGEMENT OPPORTUNITIES FOR CARE PARTNERS OF PERSONS WITH DEMENTIA

**DOI:** 10.1093/geroni/igad104.1754

**Published:** 2023-12-21

**Authors:** George Mois, Elizabeth Lydon, Sarah Jones, Ruchira Rao, Wendy Rogers, Raksha Mudar, Minakshi Raj

**Affiliations:** University of Illinois Urbana Champaign, Champaign, Illinois, United States; University of Illinois Urbana-Champaign, Champaign, Illinois, United States; University of Illinois-Urbana Champaign, Champaign, Illinois, United States; University of Illinois Urbana-Champaign, Champaign, Illinois, United States; University of Illinois Urbana-Champaign, Champaign, Illinois, United States; University of Illinois Urbana-Champaign (UIUC), Champaign, Illinois, United States; University of Illinois Urbana Champaign, Champaign, Illinois, United States

## Abstract

Care partners of persons with dementia provide critical support towards meeting the needs of their care recipients. Despite the increasing number of family care partners and their growing responsibilities related to caregiving activities, resources available to support their personal needs are limited (e.g., respite from caring, social engagement). Limited opportunities for social engagement can impact care partners’ sense of social connectivity, and ability to maintain their social network. Video technologies present an opportunity to help facilitate social engagement among care partners from the comfort of their homes, however, this area of research is understudied. The goal of our study was to understand the needs and preferences of care partners related to a video technology-based social engagement program. We conducted two focus groups (N=6) with care partners aged 60 or older with experience supporting an family member with dementia for at least six months. Participants watched a demonstration of a social engagement event and engaged in semi structured focus group interviews. We solicited their insights about their needs and preferences, perspectives on utilizing a video technology platform and their attitudes toward the proposed program. Our findings indicate that they see value in participating and are interested in using video technology as long as the program covers diverse topics of interest, there is flexibility in scheduling, and sufficient technology support is offered. The feedback provided by the focus group participants informed the optimization of the program for implementation in a pragmatic clinical trial.

